# The immunoregulatory role of gut microbiota in the incidence, progression, and therapy of breast cancer

**DOI:** 10.3389/fcimb.2024.1411249

**Published:** 2024-07-05

**Authors:** Kaiyang Xue, Jiawei Li, Ruijie Huang

**Affiliations:** State Key Laboratory of Oral Diseases, National Center for Stomatology, National Clinical Research Center for Oral Diseases, Department of Pediatric Dentistry, West China Hospital of Stomatology, Sichuan University, Chengdu, Sichuan, China

**Keywords:** gut microbiota, breast cancer, therapy, immunoregulatory, regulatory

## Abstract

Breast cancer (BrCa) is the most prevalent malignant tumor in women and one of the leading causes of female mortality. Its occurrence and progression are influenced by various factors, including genetics, environment, lifestyle, and hormones. In recent years, the gut microbiota has been identified as a significant factor affecting BrCa. The gut microbiota refers to the collective population of various microorganisms in the human gastrointestinal tract. Gut microbiota is closely associated with human health and disease development, participating in crucial physiological functions such as digestion, metabolism, immune response, and neural regulation. It has been found to influence the occurrence and treatment of BrCa through a variety of mechanisms. This article aims to review the immunomodulatory role of the gut microbiota in the development and treatment of BrCa.

## 1 Introduction

The mammalian microbiota encompasses trillions of microorganisms, comprising viruses, bacteria, archaea, and eukaryotes, residing internally and externally in the body. These microorganisms are distributed in various regions, including the skin, oral mucosa, vaginal mucosa, and conjunctiva, with the majority inhabiting the gastrointestinal system. The gut microbiota play apivotal role in food digestion, provision of essential nutrients to the host, safeguarding against pathogenic invasions, and regulating the growth and differentiation of gut epithelial cells. The interaction between the host and the metabolites generated by the microbiota itself plays a role in the host’s physiologic and pathologic changes (Sittipo et al., 2018). The gut microbiome harbors approximately 1,000 times more genes than the human genome (with over 220 million genes in the gut microbiome compared to the 23,000 genes in the human genome) (Tierney et al., 2019). Factors such as breastfeeding, solid food intake, and hormonal changes significantly impact the establishment and composition of gut microbiota (Rea et al., 2018). The diverse bacterial genera in specific regions (such as the stomach, ileum, and colon) also influence the gut immune system (Terrisse et al., 2021). Much evidence has approved that the microbiota, or local microbiota communities, profoundly affect the occurrence of cancers and the body’s response to them (Hosonuma et al., 2023). However, research on the relationship between gut microbiota and cancers, particularly BrCa, is limited.

Breast cancer (BrCa) is a type of cancer that occurs in the glandular epithelial tissue of the breast, and its incidence has been increasing over the years and tends to affect younger individuals (Li et al., 2022). The exact causes of this phenomenon are not yet fully understood. Researches has indicated that the age of menarche and menopause, lactation, changes in hormone levels, stress, and poor lifestyle have an impact on the risk of BrCa. Notably, these factors were also associated with alterations in the gut microbiome, suggesting that the gut microbiome plays a role in the progression of BrCa (McKee et al., 2021). It is reported that compared with normal people, the overall abundance of gut microbiota in BrCa patients is lower, and their metabolites are significantly different (Teng et al., 2021). Different gut microbiota and their metabolites have different effects on BrCa through distinct metabolic pathways. For example, the lipopolysaccharide biosynthesis pathway is activated in patients with malignant BrCa, while the spore production in benign patients is significantly increased (Yang et al., 2021). Besides, the microbiota can influence the development, metastasis, and management of BrCa through various biological processes, including estrogen metabolism regulation, DNA damage, and bacterial metabolite synthesis (Sampsell et al., 2020). Therefore, this review aims to provide a narrative overview of the studies related to the gut microbiota in BrCa, discussing the immunomodulatory role of the gut microbiota in disease progression and its potential applications in the treatment of BrCa.

## 2 Gut microbiota profile in BrCa

When the complex micro-ecosystem in the gut is dysfunctional, the microbiota with lower stability, diversity, and higher pathogenicity will reshape, which would change the normal physiological processes of the human body and lead to various pathological conditions (El-Sayed et al., 2021). Results from an analysis of gut microbiota in the TwinsUK cohort in the UK demonstrate an association between the incidence of BrCa and “disruptive” or non-healthy microbiota profile, implying that gut microbiota may serve as a diagnostic biomarker and treatment for BrCa (Jackson et al., 2018). Studies have found that postmenopausal BrCa patients have an enrichment of 38 bacteria, including Escherichia coli, Klebsiella sp_1_1_55, Prevotella amnii, Enterococcus gallinarum, Actinomyces sp. HPA0247, Shewanella putrefaciens, and Erwinia amylovora; however, Eubacterium_eligens, Escherichia_albertii, Campylobacter_concisus, unclassified_Enterobacteriaceae_bacterium_9_2_54FAA, Roseburia_inulinivorans, Brucella_melitensis and Lactobacillus_vaginalis were less abundant (Zhu et al., 2018). In another study by Goedert et al, the gut microbiota profile of the same subtype of BrCa patients (postmenopausal BrCa) is different from TwinsUK cohort, with an increase in the relative abundance of Clostridia, Ruminococcaceae, and Bacteroides, and a decrease in the relative abundance of Actinobacteria and the Coriobacteriaceae family (Goedert et al., 2015). The results of this study align with the findings from the TwinsUK cohort, which suggest that postmenopausal BrCa patients exhibit altered microbiota profiles, although the specific reported species may differ. Additionally, Guan et al. conducted a microbiota analysis on HER2-positive metastatic BrCa patients receiving scheduled capecitabine therapy and standard chemotherapy, revealing significant differences in the gut microbiota profiles between the two groups. In metastatic BrCa patients receiving capecitabine therapy, Blautia obeum, and Slackia showed a significant predictive correlation (Guan et al., 2020). Wu et al. assessed the composition of these patients’ microbiota based on tumor characteristics in BrCa patients. They noted that HER2-positive patients had lower alpha diversity, and the levels of Erysipelotrichaceae were lower, while levels of Clostridium and Veillonella were higher in patients with higher tumor grades. Both Veillonella and Erysipelotrichaeceae have been reported to be associated with inflammation (Kaakoush, 2015; Yu et al., 2017; Wu et al., 2020). A recent study of the effects of breast tumor implantation, growth, and removal on fecal bacterial composition and gut barrier function in mice found that impaired colon barrier integrity was associated with changes in fecal bacterial profiles in mice with tumors. For example, the relative abundance of lactobacillus was reduced and that of Bacteroides was higher in BrCa. In addition, the BrCa showed systemic inflammation responses, including splenomegalia, elevated bacterial load in the spleen, and elevated splenic and proencephalitic cytokines. 16S rRNA gene amplification of several bacteria cultured from the spleen and the matched fecal samples, suggesting that they originated in the gut. These results indicate that gut microbiota profiles were changed and gut barrier function was decreased in breast tumors, which further suggests that gut microbiota are closely related to the occurrence and development of BrCa (Loman et al., 2022).

## 3 The mechanisms of how gut microbiota regulate BrCa development

**Figure 1:**
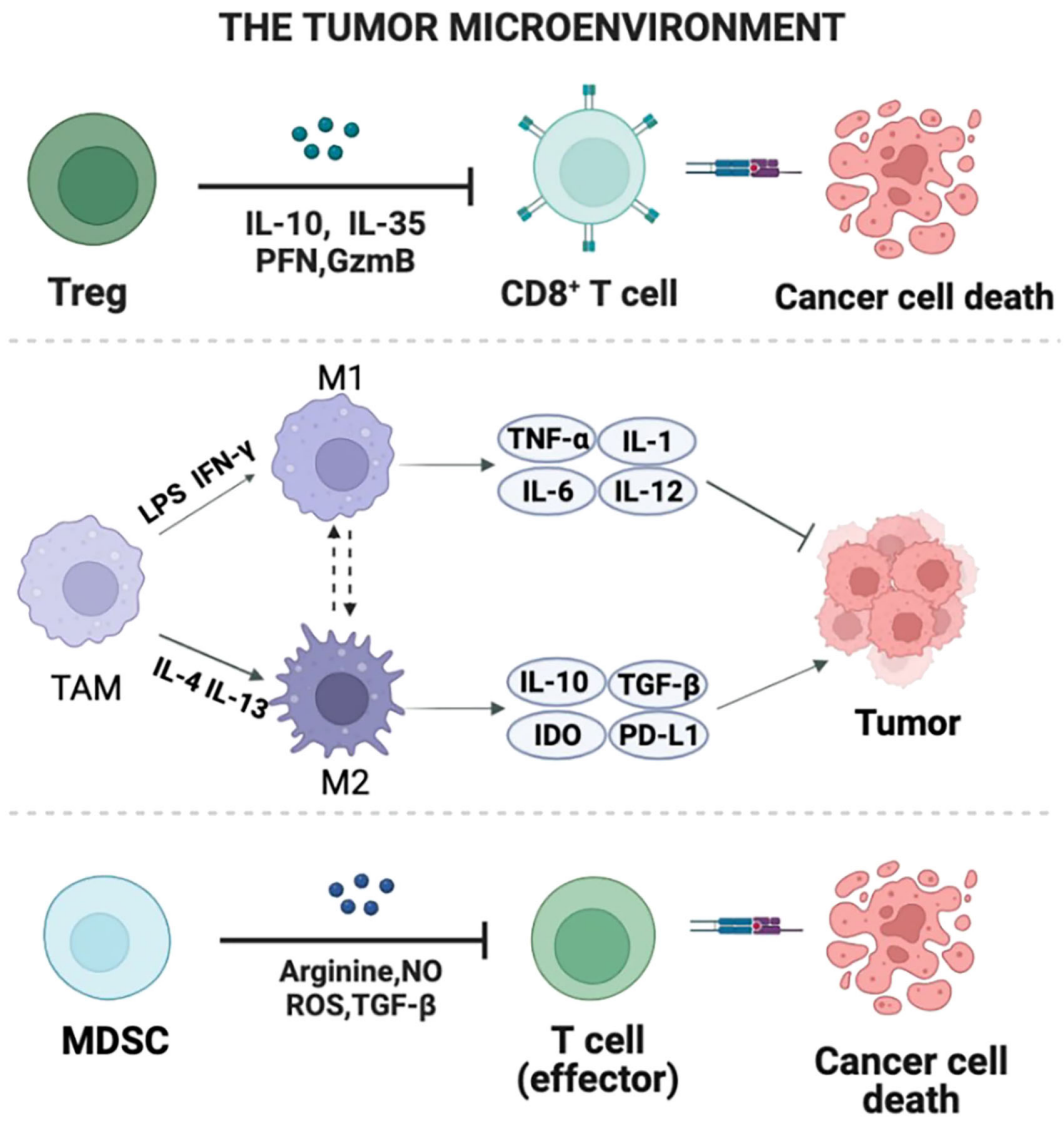
The composition of TME. Multiple chemokines and cytokines that are secreted by cancerous cells induce immune cells such Tregs, TAMs, and MDSCs to colonize the tumor. This figure reveals the regional production of immunosuppressive cytokines, chemokines, and growth factors as well as their interaction with TME components.

The gut microbiota maintains a symbiotic relationship with the host. A normal symbiotic relationship is beneficial to maintaining human health, but diet, antibiotic administration, and pathogen invasion will lead to changes in the composition of the individual microbiota, resulting in the destruction of the host immune system (Wu and Dai, 2017). Various chemokines and cytokines secreted by cancer cells induce immune cells such as regulatory T cells (Tregs), TAMs, and MDSCs to colonize the tumor (**Figure 1**) (Donald and Finlay, 2023). There is growing evidence that some specific microbiota in the gut microbiota can play a role in reducing systemic inflammation and shaping innate and adaptive immunity. The diversity and composition of gut microbiota may influence the occurrence, treatment and prognosis of BrCa patients by modulating the immune response (Lee et al., 2013; Denton et al., 2018). The effects of gut microbiota on the immune system of BrCa patients are mainly manifested in inducing the proliferation and differentiation of regulatory T cells, inducing the expression of secretory immunoglobulin (IgA), and promoting the production of neutrophils.

### 3.1 Induce proliferation and differentiation of regulatory T cells

Transcriptomic and clinicopathological information of a total of 6050 patients in three large open primary BrCa cohorts (GSE96058, METABRIC, TCGA) and 16S rRNA gene sequence microbiome data of BrCa tissues in TCGA also found the activation of cancer immunity and infiltration of anticancer immune cells in low bile acid metabolism group enriched with Lactobacillus, Ruegeria, and Marichromatium (Wu et al., 2022). It is proposed that BrCa metabolism-related microorganisms are related to rejection and immunotherapy response of Tregs and activated NK cells (Chen et al., 2023). The imbalance between increased Th17 cells and decreased Tregs leads to local and systemic inflammatory responses (Ma et al., 2020). Some beneficial human gut microbes, such as bifidobacterium and lactic acid bacteria, can promote the differentiation and function of Tregs by increasing the production of SCFAs, thereby inhibiting chronic inflammation and autoimmune responses (Markowiak-Kopeć and Śliżewska, 2020). Conversely, some harmful human gut microbes, such as Escherichia coli, Serrella, can increase chronic inflammation and autoimmune responses by producing endotoxins or inducing Th17 cell differentiation and function (Zhang et al., 2023). According to a recent study, Pseudomonas aeruginosa produces the metabolite adenosine, followed by tumor-infiltrating IFN-γ+ CD4+ and IFN-γ + CD8+ T cells, enhancing immune therapy response, and improving the efficacy of checkpoint blockade immunotherapy (Mager et al., 2020). Currently, various cancers show a close link with some bacteria, including Faecalibacterium, Lachnospiraceae, Bacteroides fragilis, Akkermansia muciniphila, Bifidobacterium, Lactobacillus, and Collinsella. Additionally, the related metabolite TMAO induces TNBC cells to undergo pyroptosis by activating endoplasmic reticulum stress kinase PERK, enhancing the *in vivo* anti-tumor immune capacity of CD8+ T cells (Li et al., 2021).

### 3.2 Induced secretory immunoglobulin (IgA) expression

Secretory IgA (sIgA) antibodies secreted by the gut form the first line of antigen-specific immune defense, preventing pathogens and symbiotic microorganisms from entering the body (Pietrzak et al., 2020). It has been reported that some bacteria in the gut microbiota can inhibit the development of tumors by activating the production of IgA, suggesting that the gut microbiota can participate in the development of tumors through IgA (Ding et al., 2021). Maternal sIgA in breast milk is known to protect newborns until the developing gut immune system begins to produce its own sIgA. The aforementioned breastfeeding is also associated with the occurrence and development of BrCa, suggesting that IgA may be a pathway that gut microbiota leads to BrCa (Sudeep et al., 2022). Some researchers have proposed that the imbalance of gut microbiota often leads to the destruction of the host immune system, and IgA protein is a potential link between BrCa-related inflammation and gut microbiota (Kaetzel, 2014). A case-control study in 2018 found that the microbiota abundance and α-diversity in IgA+ patients were significantly lower compared to IgA- patients. This suggests that the gut microbiota may influence the occurrence and development of BrCa by altering the immune pathways (Goedert et al., 2018). However, there is currently limited research on the specific mechanisms by which IgA in the gut microbiota leads to the development of BrCa, necessitating further exploration and potentially providing new therapeutic targets.

### 3.3 Promotion of neutrophil production

It is reported that neutrophils are necessary for gut bacteria to promote cancer in remote areas such as the mammary gland in mice, once again demonstrating the role of immune cells in tumor development (Hajjar et al., 2023). Neutrophils and lymphocytes are also involved in the regulation of BrCa immune response, and studies have found that high NLR (neutrophil-to-lymphocyte ratio) is associated with poor OS and DFS in BrCa patients (Ethier et al., 2017). Notably, both neutrophils and lymphocytes are regulated by the gut microbiome. In studying how the gastrointensital microbiome regulates cancer development in distant parenteral tissue, researchers infected mice with targeted pathogenic gut microbes with BrCa susceptibility, and the disease progressed significantly faster in mice with extensive neutrophilic infiltration than in mice with neutropenia. Further consumption of mouse neutrophils with the anti-LY-6GG antibody system can prevent tumor progression, suggesting that neutrophil-mediated immune response may affect BrCa progression (Lakritz et al., 2015).

## 4 Role of human gut microbiota in BrCa treatment

With the deepening of research on the relationship between gut microbes and BrCa, therapeutic methods based on the mechanism of action have emerged, such as drug metabolism, immune regulation, and the application of probiotics.

### 4.1 Modulation of drug metabolisms

The interaction between gut microbiota and drug efficacy is bidirectional. On one hand, drugs can alter the gut microenvironment, affecting the growth, composition, and functionality of bacteria. On the other hand, gut microbiota can modify drug structure through enzymatic processes, thereby changing their bioavailability, biological activity, or toxicity, resulting in individual variations in response to specific drugs (Weersma et al., 2020). It has also been observed that the presence of bacteria from the phylum Bacteroidetes or Bifidobacterium can decrease the occurrence of colitis after treatment with ipilimumab. Probiotic supplements containing Bifidobacteria also can alleviate mucositis induced by chemotherapy and diarrhea caused by radiation therapy (Badgeley et al., 2021). In a study by Di et al., the impact of gut microbiota on immune-mediated antitumor effects of trastuzumab was investigated. The study revealed that the antitumor activity of trastuzumab in mice was compromised due to antibiotic administration or fecal microbiota transplantation from antibiotic-treated donors, underscoring the direct relationship between gut microbiota and the efficacy of trastuzumab (Di Modica et al., 2021). Mendez et al. conducted an animal study and found that milk fermented by Lactobacillus casei CRL431 (PFM) reduced the side effects of capecitabine therapy for BrCa and mitigated the toxicity of capecitabine on 4T1 cells through improved immune cell stimulation induced by-PFM (Méndez Utz et al., 2021). Research on the interaction between gut microbiota and drug treatment in BrCa is relatively scarce, emphasizing the need for further investigations to comprehend how gut microbiota interacts with anticancer drugs. This will enable interventions to modulate gut microbiota for optimal therapeutic outcomes.

### 4.2 Modulation of immune responses

**Figure 2:**
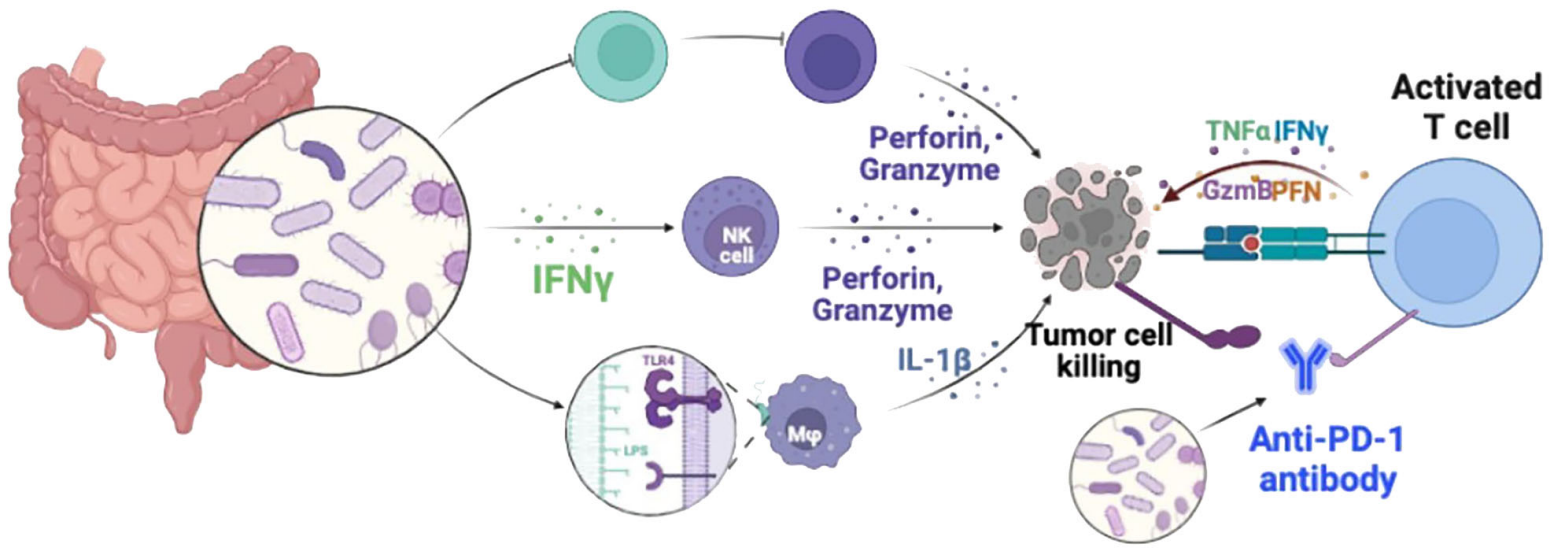
Diagrammatic illustration of immune checkpoint blockade therapies. PD-1 and PD-L1 expressed on the surface of activated T cells are the main ways to regulate the activity of T cells. Under normal physiological conditions, the main function of PD-1/PD-L1 inhibitory co-stimulatory signals is to prevent T cells from attacking normal cells through uncontrolled over-activation. However, high levels of PD-L1 are expressed on the surface of different types of tumor cells. These overexpressed PD-L1 induce T cell failure through the PD-1/PD-L1 signaling pathway, enabling tumor cells to escape T cell immune attack. PD-1/PD-L1 pathway blockers newly activate exhausted T cells in tumor immune microenvironment by blocking the PD-1/PD-L1 signaling axis, and promote the killing function of tumor cells. Restore the body’s anti-tumor immunity.

Immune checkpoint inhibitors have emerged as effective therapeutic options for various types of cancer, aiming to enhance the body’s immune capabilities to target and eliminate tumor cells (Zheng et al., 2023). Programmed cell death 1 protein (PD-1), a specific cell membrane protein associated with tumor development, disrupts the ligand-receptor interaction of PD-1/PD-L1 and inhibits the activation of the PD-1/PD-L1 signaling pathway, thereby reversing T cell exhaustion and halting immune responses within the tumor microenvironment (**Figure 2**) (Han et al., 2020). Immunotherapies such as anti-PD-1 inhibitors have become a viable treatment approach for TNBC. However, clinical evidence suggests that combining immune checkpoint inhibitors (ICIs) and NAC can improve the prognosis of early-stage TNBC but may significantly increase toxicity, thereby limiting the utility of this immunotherapeutic approach for patients (Sternschuss et al., 2021).

Preclinical murine models indicated that the gut microbiota modulates tumor responses to checkpoint blockade immunotherapy (Zhou et al., 2021). Studies on the oral and gut microbiome of melanoma patients receiving anti-PD-1 immunotherapy have found that the diversity and relative abundance of the Lachnospiraceae family in responsive patients were higher, while the germ-free mice lacking gut commensal bacteria exhibited defects in both innate and adaptive immune system (Gopalakrishnan et al., 2018). Another study found that mice treat with symbiotic bacteria Bacteroides and Bifidobacteria, in conjunction with CTLA-4 and PD-1 inhibitors, suppressed tumor growth, suggesting a correlation between gut microbiota and the effectiveness of ICIs (Elkrief et al., 2019). Extensive preliminary research indicates that specific microbiota compositions may contribute to the efficacy of immunotherapy. According to Routy and colleagues’ study using samples from patients undergoing PD-1 inhibitor therapy, fecal microbiota transplantation (FMT) from responders improved the anti-cancer effects of PD-1 blockade. Patients with low levels of Akkermansia muciniphila, a low-viscosity mucin-producing bacterium, exhibited poorer clinical responses to ICIs, indicating that bacterial supplementation may enhance immunotherapy outcomes (Routy et al., 2018). These researches suggest that gut microbiota regulation may be a new strategy to improve tumor immunotherapy. Further research is needed to elucidate how the microbiota influences BC patients’ reactions to ICIs.

### 4.3 Probiotic supplementation

Probiotics are living microorganisms that, when administered in sufficient quantities, confer health benefits to the host, possessing the capacity to regulate gut microbiota and maintain ecological balance within the ecosystem. Commonly used probiotics in products belong to genera such as Lactobacillus, Bifidobacterium, Streptococcus, Enterococcus, and Lactococcus (Zhang et al., 2021). Scholars have conducted preliminary studies on the potential efficacy of probiotic therapy for BrCa involving different genera. These studies employed cytotoxicity assays and analyses of biological markers related to proliferation and inflammation. A study reported in the internationally renowned journal Nature Medicine demonstrates for the first time that oral probiotic drug preparations can improve intestinal microbiota homeostasis in cancer patients and enhance patients’ immunotherapy response (Dizman et al., 2022). One study conducted by Imani Fooladi et al. found that compared to the control group, the survival time of BrCa mice was significantly extended in the group treated with Lactobacillus acidophilus (Imani Fooladi et al., 2015). Similarly, additional animal research affirmed the anticancer activity of orally administered Lactobacillus acidophilus in mice with breast tumors (Behzadi et al., 2021). Marschalek et al. conducted a study involving postmenopausal BrCa patients receiving chemotherapy. They were randomly assigned to an intervention group receiving four types of probiotic capsules or a control group receiving a placebo. This intervention was administered twice daily for two weeks. The study discovered that oral probiotic formulations could potentially improve the vaginal microbiota of BrCa patients undergoing chemotherapy (Marschalek et al., 2017). This evidence provides a positive direction for utilizing probiotics in treating BrCa.

## 5 Challenges and further optimization strategies in BrCa microbiota therapy research

While research on the relationship between the gut microbiota and the development of BrCa has been ongoing over the past decade, there remain many unanswered questions about the involvement mechanisms of specific bacterial strains in cancer development. These include the role of probiotics as adjunct therapy in traditional treatments, as well as the interactions between bacterial strains and the host. However, there are great differences in diet structure, living environment and genetic background of patients, resulting in certain individual differences among residents in different regions. The pathogenesis of BrCa is complex, and BrCa patients often face serious impaired immune function or neutropenia, which may affect the selection and effectiveness of gut microbiota treatment for BrCa. Therefore, more research is needed to determine the causality between BrCa and the microbiota. Furthermore, a thorough analysis of the models used, their clinical relevance, factors that may influence the microbiota (such as antibiotics and diet), and methods for integrating microbiota and host components is crucial for in-depth research in this field. In the field of cancer and immune diseases, tweaking the gut microbiota to improve efficacy is one of the hot research directions, and personalized microbiota therapy is likely to be a future treatment strategy. The aforementioned findings could pave the way for unique prognostic biomarkers for BrCa patients and improved immunotherapeutic approaches, leveraging the human microbiota to maximize the effectiveness of immunotherapy while minimizing over-treatment and negative impacts. Hopefully, more and more patients rely on regulating their own immune function to BrCa, and there are better treatment options.

## 6 Discussion

BrCa has become the most common malignant tumor worldwide, with the second highest mortality rate among female malignancies, posing a serious threat to women’s health and quality of life. Current treatments for BrCa have certain limitations and face resistance. While there is still much to be understood about the relationship between gut microbiota and BrCa, the dysregulation of gut microbiota has been identified as a crucial factor in the initiation, progression, and metastasis of BrCa. The gut microbiota can modulate the body’s immune function by influencing the proliferation and differentiation of regulatory T cells, the expression of sIgA, and the production of neutrophils, thereby impacting the onset and progression of BrCa. Numerous studies have indicated that targeted drug metabolism, altered immune regulation, and the use of probiotics may enhance treatment outcomes for BrCa patients. However, further in-depth investigations involving larger cohorts and different subtypes of BrCa are necessary to fully explore the potential application of gut microbiota as a promising intervention protocol for the clinical management of BrCa.

## Author contributions

KX: Conceptualization, Investigation, Methodology, Writing – original draft. JL: Conceptualization, Investigation, Writing – original draft. RH: Conceptualization, Funding acquisition, Project administration, Supervision, Writing – review & editing.
